# Fine-Needle Aspiration Cytology (FNAC)-Based Diagnosis of Disseminated Tuberculosis in an HIV-Positive Patient Presenting With Pyrexia of Unknown Origin

**DOI:** 10.7759/cureus.101049

**Published:** 2026-01-07

**Authors:** Santosh Kannan, Rajshree Ramasethu, Madhu Kumar

**Affiliations:** 1 Internal Medicine, MVJ Medical College and Research Institute, Bengaluru, IND; 2 Pathology, MVJ Medical College and Research Institute, Bengaluru, IND

**Keywords:** case report, cytology, diagnostic challenge, disseminated tuberculosis, extrapulmonary tuberculosis, fnac, hiv, immunocompromised host, lymphadenopathy, pyrexia of unknown origin

## Abstract

Pyrexia of unknown origin (PUO) in immunocompromised patients presents a significant diagnostic challenge, particularly when common clinical clues are absent. In human immunodeficiency virus (HIV)-positive individuals, tuberculosis (TB) often manifests atypically, leading to diagnostic delays and potentially worsening outcomes. We report the case of a middle-aged HIV-positive man who presented with persistent high-grade fever without an identifiable source. Despite comprehensive investigations, routine diagnostic tests yielded inconclusive results. Fine-needle aspiration cytology (FNAC) of an axillary lymph node revealed necrotizing granulomatous inflammation consistent with tuberculosis. The initiation of anti-tuberculosis therapy (ATT) led to rapid clinical improvement. This case highlights the importance of considering disseminated TB in the differential diagnosis of PUO in HIV-infected patients, even in the absence of classical pulmonary or systemic signs. FNAC proved to be a pivotal diagnostic tool in identifying the etiology and guiding effective treatment. Early recognition and timely intervention can markedly improve outcomes in such complex presentations.

## Introduction

Pyrexia of unknown origin (PUO) poses a diagnostic dilemma, especially in immunocompromised patients such as those with human immunodeficiency virus (HIV) infection. Tuberculosis (TB) is a known opportunistic infection in People Living with HIV (PLHIV) but often presents atypically, particularly in disseminated forms. This report illustrates the importance of considering extrapulmonary TB in PUO cases and highlights the diagnostic value of fine-needle aspiration cytology (FNAC) in detecting TB in peripheral lymphadenopathy [1].

## Case presentation

A 41-year-old male migrant worker from West Bengal, India, diagnosed with human immunodeficiency virus (HIV) infection in 2017, presented with a 2-month history of persistent high-grade fever without an identifiable source. He reported associated symptoms, including chills, odynophagia, anorexia, tingling sensations in the lower limbs, intermittent dry cough, burning micturition, and epigastric discomfort. His medical history was negative for hypertension, asthma, tuberculosis, thyroid disorders, or diabetes mellitus. He had been previously registered at an antiretroviral therapy (ART) center, but adherence to ART had been poor.

On physical examination, the patient appeared pale. Vesicular lesions with an erythematous base were noted over the hard palate, without pseudomembranous plaques suggestive of oral candidiasis. Vital signs were as follows: pulse rate 96 beats per minute, blood pressure 100/70 mmHg, respiratory rate 16 breaths per minute, oxygen saturation 97% on room air, and random blood glucose 97 mg/dL. General examination revealed multiple palpable lymph nodes in the inguinal, bilateral axillary, and right supraclavicular regions, with the largest lymph node measuring approximately 2.5 cm in the right axilla. Abdominal examination showed mild tenderness in the right hypochondrium and epigastric region, with a palpable spleen tip and a liver span of 13 cm.

Initial laboratory investigations confirmed HIV seropositivity by enzyme-linked immunosorbent assay (ELISA) and revealed a CD4 count of 105 cells/µL. Antiretroviral therapy was restarted, along with co-trimoxazole prophylaxis, after baseline evaluation, with close monitoring for immune reconstitution inflammatory syndrome (IRIS). Routine investigations, including complete blood count, urinalysis, and blood cultures, were non-contributory. Chest radiography did not reveal focal consolidation, cavitation, or miliary changes suggestive of pulmonary tuberculosis. Sputum examination for acid-fast bacilli and cartridge-based nucleic acid amplification test (CBNAAT/Xpert MTB/RIF) were negative.

Odynophagia was attributed to inflammatory mucosal lesions over the hard palate, likely representing viral or nonspecific mucositis in the setting of advanced immunosuppression. The patient also reported transient dysuria; genital examination did not reveal ulcers or discharge, urinalysis and urine culture were non-contributory, and symptoms resolved with supportive care.

**Figure 1:**
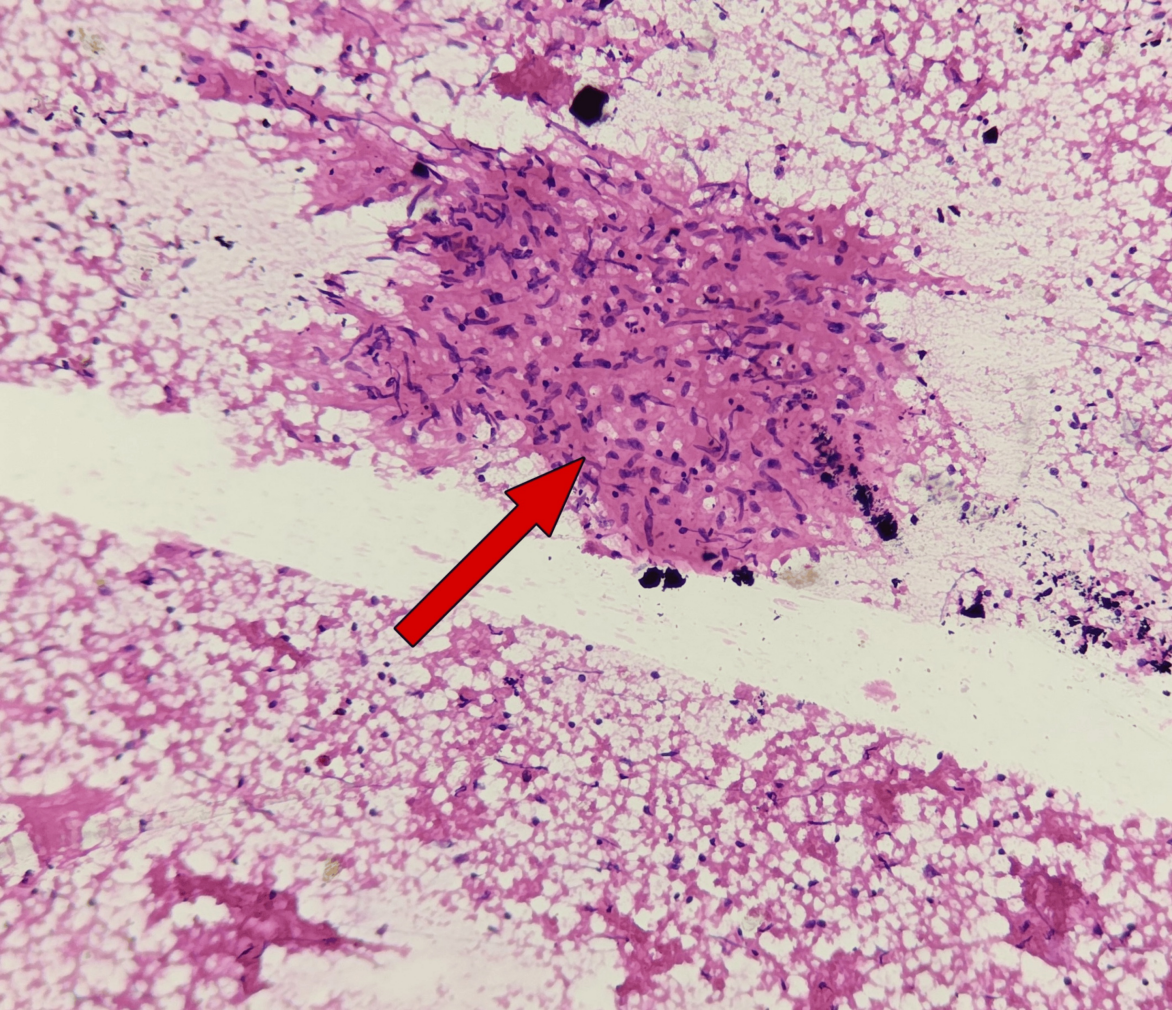
FNAC of the right axillary lymph node showing granuloma with caseous necrosis Fine-needle aspiration cytology (FNAC) of the right axillary lymph node showing necrotizing granulomatous lymphadenitis. The red arrow indicates the area of necrosis surrounded by granulomatous inflammation.

In view of prolonged undifferentiated fever in a migrant worker from a malaria-endemic region, empirical artesunate was initiated as per institutional protocol while awaiting investigations; malaria was subsequently ruled out. FNAC of the right axillary lymph node demonstrated necrotizing granulomatous lymphadenitis, consistent with tuberculosis (Figure 1).

Anti-tuberculosis therapy (ATT) was initiated immediately. The patient showed clinical improvement, with defervescence of fever and gradual weight gain. He was counseled on the chronic nature of both HIV and tuberculosis and was scheduled for regular follow-up at the Medicine Outpatient Department (OPD), Integrated Counselling and Testing Centre (ICTC), and National Tuberculosis Elimination Program (NTEP) center. No features of immune reconstitution inflammatory syndrome (IRIS) were observed during the treatment course.

## Discussion

The diagnosis of disseminated TB in HIV-infected individuals presents significant challenges, primarily due to the nonspecific nature of symptoms and the limitations of conventional diagnostic methods. In many cases, traditional investigations, including blood cultures, chest X-rays, and sputum microscopy, may fail to identify TB, particularly when the disease is extrapulmonary. In the case presented, standard diagnostic modalities were unable to provide conclusive evidence of TB despite the patient’s clinical manifestations, which raised suspicion of an infectious etiology [2].

FNAC emerged as a pivotal diagnostic tool in this case. The procedure revealed necrotizing granulomatous inflammation, a hallmark of TB, which led to a definitive diagnosis of disseminated tuberculosis. This allowed for the timely initiation of appropriate ATT, preventing further complications and improving the patient’s prognosis. FNAC’s role in diagnosing extrapulmonary TB has been well-documented in previous studies, particularly in HIV-positive individuals, where immunosuppression often leads to atypical or masked presentations of TB. In such cases, FNAC provides a minimally invasive yet highly effective diagnostic approach, offering direct access to affected tissues for histopathological examination.

The utility of FNAC in this case emphasizes its importance in the diagnostic workup of suspected TB in HIV patients, especially when conventional methods fail. It serves as a crucial tool for early detection, enabling clinicians to initiate treatment before the disease progresses to more severe stages. This case aligns with several studies that have demonstrated FNAC’s diagnostic accuracy and reliability in identifying extrapulmonary TB, thus supporting its inclusion in standard diagnostic protocols for TB in immunocompromised patients [3-5]. Timely diagnosis through FNAC can significantly reduce morbidity and mortality, underscoring its value in clinical practice.

## Conclusions

Disseminated tuberculosis (TB) should always be considered in human immunodeficiency virus (HIV)-positive patients presenting with pyrexia of unknown origin (PUO), as its clinical manifestations can be highly variable and often nonspecific. Fine-needle aspiration cytology (FNAC) of lymph nodes offers a reliable, minimally invasive diagnostic modality that can facilitate early diagnosis, thereby enabling the timely initiation of effective treatment. The successful use of FNAC in this case underscores its potential in diagnosing extrapulmonary TB, particularly in immunocompromised patients. Awareness of atypical presentations, coupled with prompt and targeted investigations, is essential for ensuring accurate diagnosis and preventing the progression of the disease. This case reinforces the importance of early intervention in improving patient outcomes in the context of HIV-associated disseminated TB.

## References

[1] World Health Organization (2023). WHO Global Tuberculosis Report. https://www.who.int/publications/i/item/9789240083851.

[2] Lawn SD, Meintjes G, McIlleron H, Harries AD, Wood R (2013). Management of HIV-associated tuberculosis in resource-limited settings: a state-of-the-art review. BMC Med.

[3] Patel A, Pundkar A, Agarwal A, Gadkari C, Nagpal AK, Kuttan N (2024). A comprehensive review of HIV-associated tuberculosis: clinical challenges and advances in management. Cureus.

[4] Samaila MO, Oluwole OP (2011). Extrapulmonary tuberculosis. Fine needle aspiration cytology diagnosis. Niger J Clin Pract.

[5] Kumbi H, Ali MM, Abate A (2024). Performance of fine needle aspiration cytology and Ziehl-Neelsen staining technique in the diagnosis of tuberculosis lymphadenitis. BMC Infect Dis.

